# Development of RP HPLC‐PDA method for simultaneous quantitative analysis of Inoscavin A and Meshimakobnol A and application on some *Phellinus* mushroom species

**DOI:** 10.1002/fsn3.4031

**Published:** 2024-02-22

**Authors:** Anh Ngoc Le, Tuan Ngoc Nguyen, Dao Thi Anh Dong

**Affiliations:** ^1^ Department of Food Technology, Faculty of Chemical Engineering Ho Chi Minh City University of Technology (HCMUT) Ho Chi Minh city Vietnam; ^2^ Vietnam National University Ho Chi Minh City (VNU‐HCM) Ho Chi Minh city Vietnam; ^3^ Institute of Biotechnology and Food Technology Industrial University of Ho Chi Minh City Ho Chi Minh City Vietnam

**Keywords:** Inoscavin A, Meshimakobnol A, *Phellinus* mushroom, RP HPLC‐PDA

## Abstract

*Phellinus igniarius*, a medicinal mushroom containing many active ingredients with health benefits, can be applied in functional food. At present, the quantification of the main active ingredients from higher fungi (*Ganoderma*, *Phellinus…*) materials from different growing sources is a mandatory requirement to standardize the input resources of pharmaceutical and food production. Our study's aims are to perfect the RP HPLC‐PDA method for quantitative analysis of Inoscavin A and Meshimakobnol A which are two main active ingredients present in *Phellinus* mushroom. In this analytical method, a C18‐HPLC column and the mixture of methanol and formic acid solutions (pH = 2.2) are used to analyze and elute the active substances with the column activity parameters being the concentration gradient. This perfect method was tested for system suitability, repeatability, intermediate precision, recovery, and linear curve calibration to validate the method. After validation, the perfected RP HPLC‐PDA method was applied to analyze eight samples of *Phellinus* and three samples of *Ganoderma mushroom category*. This method can be the basis for classifying between *Phellinus* and some other medicinal mushrooms.

## Graphical abstract

In this research, the method for simultaneous quantification of Inoscavin A and Meshimakobnol A was investigated. The results indicated this method was suitable for both compounds through validation using repeatability, linearity, recovery, and intermediate precision and applied to analyze several species of *Phellinus* mushroom. This method could be the basis for classifying between *Phellinus* and some other medicinal mushrooms.

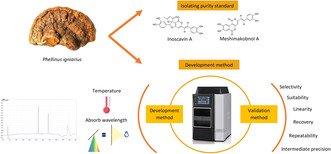

## 1 INTRODUCTION

*Phellinus*, a higher fungus belonging to the Hymenochaetaceae family, has been a well‐known oriental medicine mushroom in Asian countries for thousands of years. Fruiting bodies that grow on wood are mainly yellowish to rusty brown or gray to black (Kawashte et al., 2021; Suabjakyong et al., 2015). In recent years, *Phellinus* has attracted worldwide attention and research efforts toward the scientific rediscovery of this traditional remedy. Previous phytochemicals research found that *Phellinus* contains secondary and biologically active metabolites such as polysaccharides, terpenoids, steroids, styrypyrone, and polyphenol, which can demonstrate medical properties including anti‐inflammation, antidiabetic, antitumor, anticancer, antioxidant, and other bioactivities (He et al., 2021; Liu et al., 2020; Luan et al., 2022; Sun et al., 2021).

Inoscavin A (1) and Phelligridin D or Meshimakobnol A (2) are naturally occurring styrylpyrone classes and were found in several species of *Phellinus* including *P. igniarius*, *P. baumii*, and *P. linteus* (Kojima et al., 2008; Lee et al., 2010; Mo et al., 2004). Both substances are phenylpropanoid‐hispidin derivatives and have free radical scavenging activity. Therefore, they are the main components having effective anticancer and anti‐inflammatory effects (Huo et al., 2020; Kim et al., 2018; Lee et al., 2010; Yoon & Paik, 2010). Previous studies focused only on reporting chemical structures and determining the crude biological activity extracted on their own extract (Dong et al., 2020; Kojima et al., 2008).

Based on DNA sequencing technologies, there were estimated from 2.2 to 3.8 million fungal species (Hyde, 2022). Therein, the data showed approximately 700 species out of 2000 safe species known having pharmacological properties (Wasser, 2017). Thanks to chemical composition and biologically active compounds having benefits for health, higher fungi crude and product's economic values have increased globally. The size of the global mushroom market was 14.35 million tons in 2022 and is estimated to attain 24.05 million tonnes in 2028. The most popular functional mushroom market comprises Reishi (*Ganoderma lucidum*), Lion's Mane (*Hericium erinaceus*), and Turkey Tail (*Trametes versicolor* (L.) Lloyd) (Łysakowska et al., 2023). *Phellinus* was considered a medicinal fungus that could be a supplement food in cancer treatments in Korea. Besides, some products originating in *Phellinus* like tea, drink, and concentrated extracts are also appreciated by Asian consumers and gradually expand the market globally, especially with those who pursue health (Zhou et al., 2022). Recently, containing *Phellinus* products which are made from one of the wild mushrooms (natural fruiting bodies), cultivated mushrooms (cultivated fruiting bodies), cultured mycelium or even their mixture. Several patents have been issued for culturing fruiting bodies or obtaining beneficial biomass from *Phellinus* demonstrating their potential in the pharmaceutical or food industry, like the publication of KR20040094497A (2003) by 박준덕, 박순영 for food material using *Phellinus linteus* and publication of CN113398153A (2021) by Anhui Limin Biological Technology Co ltd for a method for utilizing *Phellinus igniarius* mycelium. Jung‐Ok Kim et al. (2008) reported the applicability of hot water extract and ethanol extract from *Phellinus linteus* by using total flavonoids content, total phenolics content, and some other bioactivity to evaluate crude extract from both solvents. The authors also claimed that the ethanol extract was the most pertinent for use as functional food (Kim et al., 2008). Jasmina Dimitrova‐Shumkovska et al. (2022) reported adding hot water extracted from *Phellinus torulosus* and *Phellinus igniarius* to yogurt products. The authors evaluated the above study has high applicability to create various functional foods appearing in *Phellinus* extract (Dimitrova‐Shumkovska et al., 2022). Meanwhile, the composition of higher fungi is influenced by various factors comprising climate change, ecological group conditions, and geographical area leading to the change in raw material quality (Vita et al., 2018). Besides, the processing method including thermal, chemical, and physical ways was also determined by the change of bioactive components (Marçal et al., 2021; Zivanovic & Buescher, 2004). The rise of products originating from *Phellinus* and the other medicine mushroom species required to development of a method of high‐performance liquid chromatography with photodiode array detection (HPLC‐PDA) to separate and analyze Inoscavin A and Meshimakobnol A to compare natural bodies, and cultured bodies, between *Phellinus* and some other mushrooms.

**Table:** 

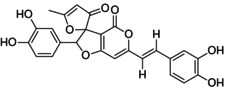	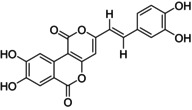
Inoscavin A (1)	Meshimakobnol A (2)

This study aims to develop an RP HPLC‐PDA method for the qualitative analysis of these two styrylpyrones. Validation of the method was performed for selectivity, linearity, stability, and repeatability. This can be applied to RP HPLC‐PDA profiling to analyze other wild and commercial mushrooms in Vietnam to provide a scientific basis for composition and use it in food or pharmaceutical food industries. The data would be valuable for calculating the contribution of those higher fungi to bioactivity in people's daily diets.

## 2 MATERIALS AND METHODS

### 2.1 Chemicals

Inoscavin A (C_25_H_18_O_9_) and Meshimakobnol A (C_20_H_12_O_8_) used for standard were isolated from *Phellinus igniarius*. Inoscavin A and Meshimakobnol A powder was stored in black glass at −20°C. Stock standard solutions were prepared in the dark and kept in a refrigerator −20°C until analysis. Water and methanol of HPLC grade were purchased from Fisher Scientific (USA), formic acid of HPLC grade were purchased from Scharlab (Spain), and ethanol and water (analytical chemicals) for extracting were purchased from Hoa Nam Chemical Co., ltd (Vietnam).

### 2.2 Prepare purified Inoscavin A and Meshimakobnol A as standards for HPLC analysis

The fruiting bodies of *P. igniarius* were collected from Puhuong National Park of Nghean Province, Vietnam, in April 2019 and identified by Prof. Dr. Ngo Anh, Department of Biology, Hue University. The purity standard was prepared according to Thanh et al. (2018) (Thanh et al., 2018). The air‐dried and powdered fruit body of *P. igniarius* (11 kg) was extracted by 98% EtOH at room temperature for 3 × 7 days. Then, the extracted mixture was removed solvent by a rotary evaporator (IKA® RV 10, Germany) under the temperature of 40°C. After removing solvents, the deep brown syrup (700 g) was suspended in water and partitioned with ethyl acetate to afford ethyl acetate fractions (56 g).

The ethyl acetate fractions (EAF) were applied to silica gel column chromatography (600 g, 160 × 7 cm) with a chloroform:methanol (C:M) step gradient system (100:0, 40:1: 30:1; 20:1; 10:1: 4:1; 2:1) to obtain seven minor fractions. This process was supervised by TLC to obtain five major fractions. Fraction 3 was eluted into the silica gel column chromatography with the mixture C:M (30:1) to afford Inoscavin A (120 mg). The Inoscavin A (120 mg) was purified by HPLC with Luna C_18_ preparative column (methanol: water 20:1; 2.0 mL/min) to obtain Inoscavin A (45 mg). Fraction 5 was eluted into the silica gel column chromatography by the mixture C:M (10:1) to get Meshimakobnol A (20 mg). The Meshimakobnol A (20 mg) was purified by HPLC with Luna C_18_ preparative column (methanol:water 4:1; 2.0 mL/min) to give Meshimakobnol A (5 mg). The diagram of isolating Inoscavin A and Meshimakobnol A purity standards is shown in Figure S1 in Supporting Information.

The purified standards was evaluated by combining nuclear magnetic resonance spectroscopy (NMR) and high‐performance liquid chromatography (HPLC). Spectroscopic data of Inoscavin A (Figures S2–S4; Table S1) and Meshimakobnol A (Figures S5–S7; Table S2) purity standard is shown in Supporting information.

### 2.3 Collection of mushroom samples

Ten fruiting bodies comprising *Phellinus nilgheriensis* (PS1), *Phellinus baumii* (PS2), *Phellinus linteus* (PS3), *Phellinus linteus* (PS4), *Phellinus pomaceus* (PS5), *Phellinus pini* (PS6), *Phellinus igniarius* (PS8), *Ganoderma applanatum* (GS1), *Ganoderma australe* (GS2), and *Ganoderma brownii* (GS3) were collected from national park in Nghe An province, Viet Nam in 2022. A fruiting body of *Phellinus igniarius* (PS7) was purchased at Linh Chi Vina joint stock company in Viet Nam in 2022. The portrait of those mushrooms is shown in Table S3 of supporting information. The entire mushrooms were identified by Prof. Dr. Ngo Anh, Department of Biology, Hue University, Viet Nam.

### 2.4 Sample preparation

#### 2.4.1 Preparation of the extracts

Fruiting bodies were ground with an electric grinder into a powder (approximately 60 mesh). They were vacuum sealed and stored in PA/PE full form at first instance in bags at −20°C until extraction. The powdered fruiting bodies were extracted according to Dokhaharani et al. (2021) (Dokhaharani et al., 2021) and modified for fit experiments. Powdered mushroom samples were weighed 10 ± 0.0001 g for ultrasonic‐assisted extraction with an ultrasonic (Vibra‐Cell VCX‐500, Sonics®, USA) for 5 min; ultrasonic power 25%; frequency 20 KHz; ethanol concentration 80% (v/v) at room temperature (30–32°C) and the volume was used 200 mL. Then, the sample was added to ethanol (the total volume of ethanol gives a sufficient volume of 300 mL) and sonicated in an ultrasonic bath (Sonica 2200 EP S3, Soltec®, Italy) at a temperature of 60°C; extract time of 280 min; frequency of 40 KHz. The sample was filtered through filter paper (Whatman®, grade 4) by a vacuum pump to divide the extract and solid. The solids were washed three times and mixed with the prior eluate before being placed in a rotary evaporator (IKA® RV 10, Germany) at a temperature of 40°C. The extract was then lyophilized (ScanVac Coolsafe 9 L, Labogene, Denmark) into a powder. The sample powder was prepared similarly to the standard solution prior to injection. The powders were stored in a refrigerator at −20°C until analysis. All tests were repeated in triplicate.

#### 2.4.2 Preparation of standards

The standard solution was prepared at the concentration range between 0.1 and 5.0 mg/L for Inoscavin A, and between 1.5 and 75.0 mg/L for Meshimakobnol A. The standard of Inoscavin A (100 mg/L) and Meshimakobnol A (100 mg/L) was dissolved by combining methanol and water with a formic acid solution (pH = 2.2) to give a stock solution for each compound. The mixture standard was prepared by mixing and diluting both of them in the flask with methanol formic acid solution (pH = 2.2) to establish a calibration curve based on six points.

### 2.5 HPLC‐PDA analysis

In this study, instrumentation for HPLC analysis consisted of a Shimadzu LC‐2030C 3D liquid chromatographic system (Shimadzu, Japan), equipped with a Photodiode array (PDA) detector. The chromatograph was equipped with a VertiSep™ GES C_18_ HPLC column (250 mm × 4.6 mm, 5.0 μm) and controller by Labsolution software.

### 2.6 Method validation

After the study of analytical parameters, the validation of our analytical basis was performed according to the guidelines of Eurachem (Magnusson & Örnemark, 2014). The method was validated through assessment of selectivity, linearity, repeatability, intermediate precision, recovery, limit of detection (LOD), and limit of quantification (LOQ). Among them, the recovery range is referenced in the Guidelines for Standard Method Performance Requirements (AOAC international, 2019).

### 2.7 Statistical analysis

The results were calculated based on triplicate trials and presented as mean ± standard deviation (SD). One‐way Analysis of Variance (ANOVA) and Multiple Range Test were used for determining the significant difference between the Inoscavin A and Meshimakobnol A concentrations among the samples (significant difference at *p* < .05). The linear regression analysis method was used to establish a calibration curve to quantify Inoscavin A and Meshimakobnol A in all samples.

## 3 RESULTS AND DISCUSSIONS

### 3.1 Method development

Inoscavin A and Meshimakobnol A belonging to the styrylpyrone classes showed characteristic maximal absorption at 380–420 and 245–255 nm (Lee et al., 2009). There have been no studies on the simultaneous quantification of Inoscavin A and Meshimakobnol A. There is only one study by Kazuo Kojima et al. (2008) about the appearance of both compounds according to retention time. Nevertheless, the results of the above study only determined the amount of Meshimakobnol A in the material without Inoscavin A. Besides, the paper did not have a standard curve area obtained and validate the method. While, the chromatographic observations of the above study also revealed differences between wild, cultivated, and commercial mushrooms not only in the composition of Meshimakobnol A but also in Inoscavin A which could be a composition to distinguish among wild and cultivated mushrooms. The other study by Dan Shou et al. (2016) (Shou et al., 2016) quantitated Inoscavin A without Meshimakobnol A which could be a differentiation profiling between *Phellinus* and other mushrooms. Besides, our experimental data shows that Inoscavin A is one of the major pigments in *Phellinus* species in Vietnam. In addition, Inoscavin A and Meshimakobnol A standard grades have not yet been available on the market which is the reason why quantification simultaneously both of them is difficult.

**FIGURE 1:**
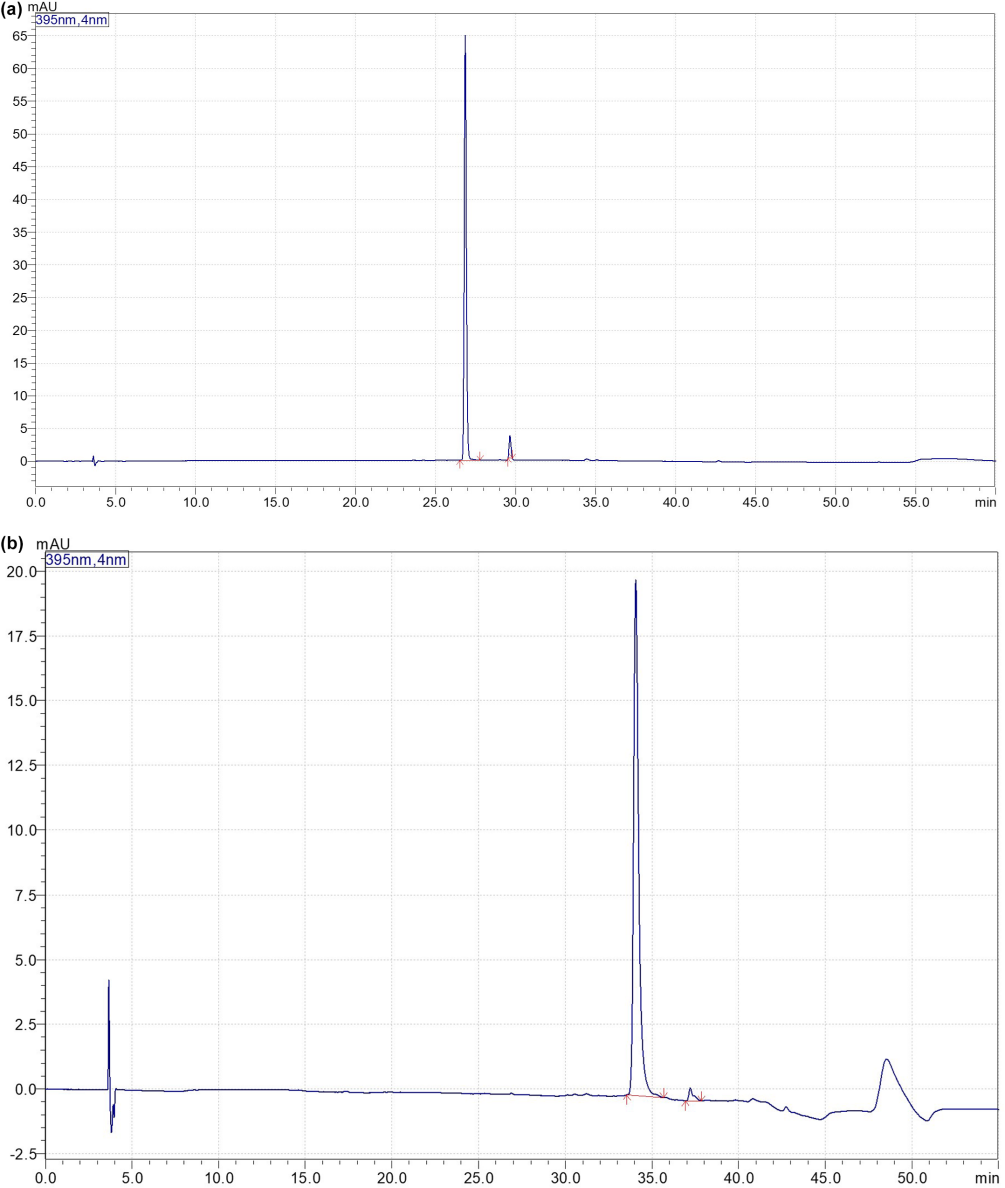
The chromatogram of purity standards (a) Inoscavin A; (b) Meshimakobnol A.

Inoscavin A and Meshimakobnol A purity standard in this study was isolated similarly to the implementation process that was mentioned in Section 2.2. The structure of the obtained powders, after being eluted into the silica gel column chromatography, was identified by using nuclear magnetic resonance spectroscopy (NMR) and compared with the old data reported before in the cited literature. Compound (1) has a structure similar to Inoscavin A which was described by Kim et al. (1999) and Thanh et al. (2016) (Kim et al., 1999; Thanh et al., 2016). Compound (2) has a structure similar to Meshimakobnol A which was described by Mo et al. (2004) and Thanh et al. (2018) (Spectroscopic data analyzed by NMR are shown in Supporting Information) (Mo et al., 2004; Thanh et al., 2018). The results (Figure 1) of purity target standards verified using HPLC showed that the purity of Inoscavin A and Meshimakobnol A reached 95.01 and 97.93%, respectively.

**FIGURE 2:**
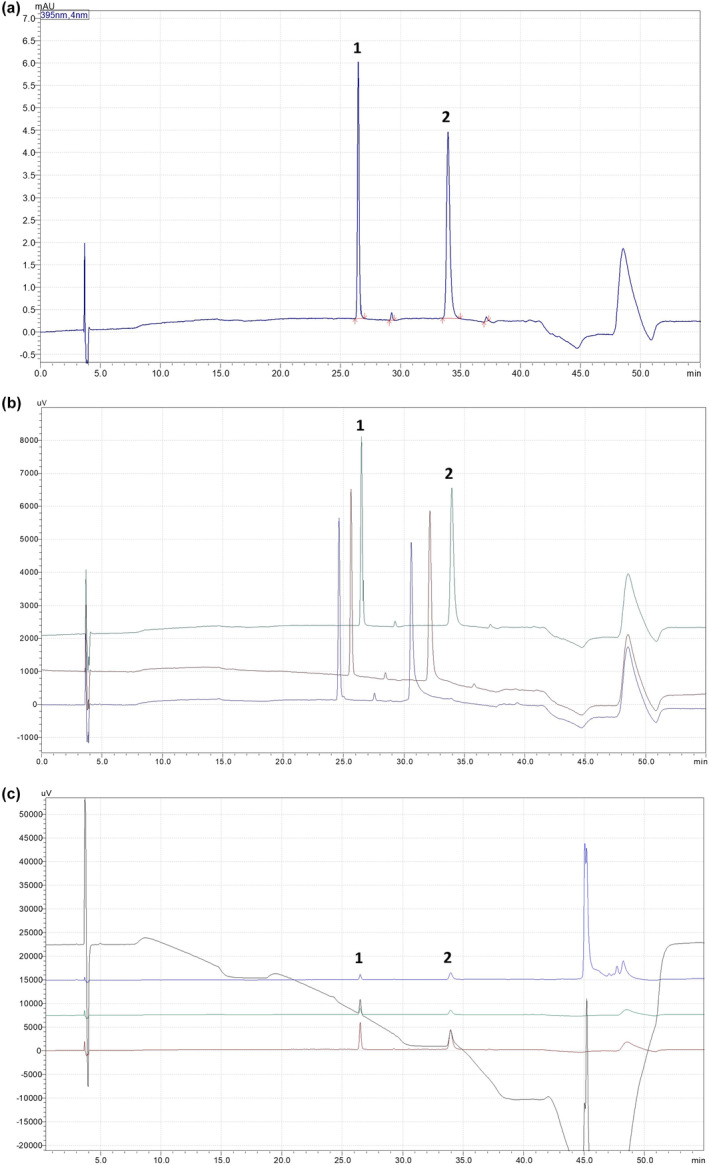
The chromatogram of Inoscavin A (1) and Meshimaobnol A (2); (a) standard solution at temperature of 30°C, flow grade 0.8 mL/min; (b) Temperature at 30°C (green), 35°C (brown), 40°C (blue); (c) Detected at 250 nm (black), 295 nm (blue), 330 nm (green), 395 nm (red).

For the gradient of the mobile phase, we studied the different types of stream using a mixture of methanol and formic acid solutions (pH = 2.2), and the parameters including temperature, flow grade, injection volume, and solvents for the mobile phase are presented in Table 1. The result in Figure 2a showed that Inoscavin A and Meshimakobnol A had high resolution and short tails in both peaks.

**TABLE 1:** The summary of HPLC‐PDA parameters for Inoscavin A and Meshimakobnol A.

Column	VertiSep™ GES C_18_ HPLC (250 mm × 4.6 mm, 5.0 μm)
Flow rate	0.8 mL/min
Injection volume	20 μL
Wavelength	395 nm
Column temperature	30°C
Mobile phase	Methanol (A): formic acid solutions pH = 2.2 (B)
Gradient elution mode	0–3 min, 25% (A), 3–10 min, 25–40% (A), 10–14, hold 40% (A), 14–25 min, 40–60% (A), 25–29 min, hold 60% (A), 29–33 min, 60–70% (A), 33–37 min, hold 70% (A), 37–40 min, 70–85% (A), 40–43 min, hold 85% (A), 43–46 min, 85–25% (A), 46–55 min, hold 25% (A)
Run time	55.00 min

The chromatograms in Figure 2b showed the effect of temperature on the elution of the substances in the stationary phase. The study was conducted at temperatures 30, 35, and 40°C (Figure 2). At 30°C, it shows separation well. While, at 35 and 40°C, peak tails formed, leading to poor separation from other peaks (Table 2).

**TABLE 2:** The asymmetry of Inoscavin A and Meshimakobnol A.

	Inoscavin A				Meshimakobnol A			
	W	W_a_	W_b_	A_sys_	W	W_a_	W_b_	A_sys_
30°C	0.333	0.147	0.186	1.265	0.721	0.267	0.454	1.700
35°C	0.335	0.134	0.201	1.500	0.637	0.218	0.419	1.922
40°C	0.208	0.115	0.093	0.809	1.105	0.181	0.924	5.105

*Note*: Where W: width of the whole peak base; W_a_: width of the 1st half peak; W_b_: width of the 2nd half peak; A_sys_: the asymmetry of peak.

The study by Dan Shou et al.&amp;#x000A0;(2016) (Shou et al., 2016) about using LC‐PAD and LC–MS methods for the rapid quantification of styrylpyrone classes showed that both of them were well suited for this work. On the other hand, Inoscavin A and Meshimakobnol A could be absorbed at a given wavelength. For simultaneously identifying and quantifying two compositions, this study had evaluated the absorb by LC Labsolution software and made a comparison with the reference, four wavelengths including 250, 295, 330, and 395 nm. The results (Figure 2c) showed that the wavelengths at 395 nm give high absorbance for both of them. This result was similar to the study of Dan Shou using wavelength at 395 nm for obtaining Inoscavin A and Hypholomine B (belongs to styrylpyrone classes).

### 3.2 Method validation

#### 3.2.1 System suitability testing

System suitability testing (SST) is to demonstrate that the analytical method is working perfectly and to ensure the quality of the method is appropriate. Therefore, conformity testing has been identified as part of the development and validation methodology. System fit was calculated by taking data from six replicates of the mixed standard solution. SST is evaluated through relative standard deviation (RDS%) for the area under the curve and retention time. The RSD value is 2% lower showing that the method is appropriate (Broadhurst et al., 2018; Miller & Miller, 2005). The result of the suitability test is shown in Table 3. The SST value of Inoscavin A is 0.47% for the mean area curve and 0.04% for retention time. Meshimakobnol A' SST value is 0.29% for the mean area curve and 0.04% for the retention time demonstrating the method is high strength.

**TABLE 3:** The results of validation method.

Parameters	Inoscavin A	Meshimakobnol A
Calibration curve area	Y = aX + b a = 59621.6 b = −694.057	Y = aX + b a = 21716.6 b = −1687.23
*R* ^2^	.9999	.9996
Linearity range (mg/L)	0.111–5.015	1.372–75.307
LOD (mg/L)	0.012	0.05
LOQ (mg/L)	0.04	0.17
Repeatability, RSD% (*n* = 9); Analyst A	0.8182	0.7577
Intermediate precision, RSD% (*n* = 9); Analyst B	1.3156	1.4236
System suitability test (*n* = 6)		
Mean area under curve, RSD%	28,695 (0.4704)	260,388 (0.2941)
Retention time, min, RSD%	26.7828 (0.0395)	34.4285 (0.0456)
Recovery* (*n* = 9), %	99.6790	99.4763

* Average of recoveries at three spike levels at 50, 100, 150% compared with the concentration of the sample.

#### 3.2.2 Calibration curve and linearity

The linearity curve calibration was established by linear regression analysis using LabSolution software (Table 3). Six concentration points of Inoscavin A and Meshimakobnol A (Table 3) were used to construct the calibration curve for each compound. The coefficient of determination (R) was used for the suitability of the linear curve calibration. The results showed that the calibration curve area for Inoscavin A was Y = 59621.6X–694.057 (*R* ^2^ = .9999) and for Meshimakobnol A was Y = 21716.6X–1687.23 (*R* ^2^ = .9996) having good accuracy is suitable for quantify both of compounds. Besides that, limit of detection (LOD) and limit of quantitation (LOQ) were used to evaluate the system sensitivity.

#### 3.2.3 Repeatability and intermediate precision

Repeatability in exact same‐day runs was evaluated by calculating the RSD (%) of triplicate injection of three samples under similar operating conditions. The intermediate precision was measured by calculating the RSD (%) of triplicate injections for three samples by different analysts repeated exactly the other day. The result is presented in Table 3.

#### 3.2.4 Determination of recovery

The trueness of the method was verified by testing the recovery experiments using spiked samples. In this study, three of the standard solution mixtures were added to the sample of definite concentrations. The average of triplicate injection for three samples adding the standard solution mixture. Results (Table 3) of recovery samples including 50% (92%), 100% (102.5556%), 150% (104.4815%) for Inoscavin A 50% (92.5741%), 100% (99.3611%), 150% (106.4938%) for Meshimakobnol A were compared and referenced in the Guidelines for Performance of Standard Methods (AOAC international, 2019) to assess recoverability of the method showed good recovery.

### 3.3 Concentration of Inoscavin A and Meshimakobnol A in several mushrooms

The method using HPLC‐PAD established and validated by us has been successfully utilized for quantifying simultaneously Inoscavin A and Meshimakobnol A from wild fruiting bodies and commercial mushrooms in Vietnam. The results of Inoscavin A and Meshimakobnol A concentration are presented in Table 4.

**TABLE 4:** Content of Inoscavin A and Meshimakobnol A in several mushrooms.

Sample	Inoscavin A			Meshimakobnol A		
	Concentration (mg/g)	SD (*n* = 3)	RSD (%)	Concentration (mg/g)	SD (*n* = 3)	RSD (%)
PS1	0.1832^a^	0.0021	1.1553	0.3083^a^	0.0010	0.3266
PS2	9.0940^g^	0.1368	1.5047	6.6267^c^	0.1773	2.6762
PS3	6.1547^d^	0.0753	1.2230	4.4273^b^	0.0586	1.3235
PS4	6.9578^e^	0.3088	4.4388	11.8811^d^	0.5117	4.3069
PS5	1.3480^c^	0.0066	0.4865	0.0000^a^	0.0000	0.0000
PS6	9.5233^h^	0.2150	2.2627	0.0000^a^	0.0000	0.0000
PS7	0.0000^a^	0.0000	0.0000	20.5600^e^	1.0630	5.1701
PS8	7.3877^f^	0.3107	4.2062	46.4300^f^	2.4116	5.1940
GS1	0.5860^b^	0.0021	0.3554	0.0000^a^	0.0000	0.0000
GS2	0.0810^a^	0.0010	1.2845	0.0000^a^	0.0000	0.0000
GS3	0.1090^a^	0.0016	1.4395	0.0000^a^	0.0000	0.0000

*Note*: The characters a, b, c, d, e, f, g, and h present a significant difference at a confidence interval of 95%.

The content of Inoscavin A and Meshimakobnol A ranged from 0.0810 to 9.5233 mg/g and 0.3083 to 46.4300 mg/g, respectively. Among these, there are some species such as PS2, PS3, PS4, PS5, PS6, and PS8 belonging to the *Phellinus* genus had higher content than *Phellinus igniarius* as reported by Dan Shou et al. (2016) originating in China (Shou et al., 2016). Meshimakobnol A content in PS2, PS3, PS4, PS7, and PS8 is higher than the result from natural fruiting bodies by Kazuo Kojima et al. (2008) (Kojima et al., 2008). These differences depend on the climatic conditions, geographical origins, habitat, and cultivated conditions (Kojima et al., 2008; Yu et al., 2016). PS3 and PS4 samples are identified as *Phellinus linteus*, therein PS4 was more perennial than PS3, which could be the reason why the content of Inoscavin A and Meshimakobnol A in PS4 is more than PS3.

The result of this study showed that Inoscavin A (1) is one of the main products from the fruiting bodies of natural mushrooms belonging to *Phellinus* and *Ganoderma* families and is not present in the commercial PS7 as *Phellinus igniarius*. Meanwhile, PS8 was also identified *Phellinus igniarius* which was collected in the wild containing high concentration Inoscavin A. This result was similar to Kazuo's study in that Inoscavin A is absent in some commercial *Phellinus linteus* samples originating in China and Korea (Kojima et al., 2008). These results showed that Inoscavin A has the potential to be used to differentiate between wild and cultivated mushroom fruiting bodies.

Several *Ganoderma* mushrooms belonging to samples including *Ganoderma applanatum* (GS1), *Ganoderma australe* (GS2), and *Ganoderma brownii* (GS3) were analyzed for the content of Inoscavin A and Meshimakobnol A. The result showed that Inoscavin A content in *Ganoderma* is lower than that in *Phellinus* species. This is the first time, Inoscavin A was determined in *Ganoderma* species in low concentrations. However, the result also showed that there is no occurrence of Meshimakobnol A in *Ganoderma* species. Meanwhile, Meshimakobnol A has appeared in many different *Phellinus* and is evaluated as one of the main pigments of the naturally growing *P. linteus* fruiting body (Kojima et al., 2008). The above results showed that Meshimakobnol A can be the basis for classifying *Phellinus*, especially natural *P. linteus* with *Ganoderma* species.

## 4 CONCLUSION

Inoscavin A and Meshimakobnol A are the two main active ingredients and two main pigments of *Phellinus* mushroom in Vietnam. They have biological activities that were shown to be beneficial to human health. The temperature at 30°C for the RP HPLC‐PDA system permitted quantification Inoscavin A and Meshimakobnol A well that was evaluated by the system parameters A_sys_ show good elution. Quantitative analytical methods for these two active ingredients have been established and analyzed on seven native and one commercial *Phellinus* species and three *Ganoderma* species. Our method can qualitatively and simultaneously analyze Inoscavin A and Meshimakobnol A in mushrooms, especially *Phellinus* species by RP HPLC‐PDA method without using a purification step. In addition, this method also contributes significantly to building fingerprints between natural *Phellinus* and commercial fruiting bodies and distinguishes *Phellinus* from others, especially *Ganoderma*. Validation of our method has been demonstrated through suitability, repeatability, intermediate accuracy, and reproducibility. For this reason, this method is suitable for the quantitative analysis of Inoscavin A and Meshimakobnol A in *Phellinus* and some other mushrooms.

## AUTHOR CONTRIBUTIONS

**Dao Thi Anh Dong:** Conceptualization (equal); investigation (equal); methodology (equal); project administration (equal); supervision (equal); validation (equal); writing – review and editing (equal). **Anh Ngoc Le:** Conceptualization (equal); data curation (equal); formal analysis (equal); investigation (equal); methodology (equal); resources (equal); software (equal); validation (equal); visualization (equal); writing – original draft (equal). **Tuan Ngoc Nguyen:** Conceptualization (equal); data curation (equal); formal analysis (equal); investigation (equal); methodology (equal); resources (equal); software (equal); validation (equal); visualization (equal); writing – original draft (equal); writing – review and editing (equal).

## CONFLICT OF INTEREST STATEMENT

The authors declare that they have no conflict of interest.

## Supporting information


Data S1.

## Data Availability

The datasets used and/or analyzed during this study are available from the corresponding author upon reasonable request.
