# Supplementary material for: Development of RP HPLC‐PDA method for simultaneous quantitative analysis of Inoscavin A and Meshimakobnol A and application on some Phellinus mushroom species
Source: Food Sci Nutr. 2024 Feb 22;12(5):3602–11. doi: 10.1002/fsn3.4031 (PMC11077241; doi:10.1002/fsn3.4031)
Supplement: Supplementary file 1 — Data S1. [file FSN3-12-3602-s001.docx]

**Supporting Information**

**Development of RP HPLC-PDA method for simultaneous quantitative analysis of Inoscavin A and Meshimakobnol A and application on some Phellinus mushroom species.**

*Le Ngoc Anh ^a,b^, Nguyen Ngoc Tuan ^c^, Dong Thi Anh Dao ^a,b*^*

*^a^Department of Food Technology, Faculty of Chemical Engineering, Ho Chi Minh City University of Technology (HCMUT), 268 Ly Thuong Kiet Street, District 10, Ho Chi Minh city, Viet Nam*

*^b^Vietnam National University Ho Chi Minh City, Linh Trung ward, Ho Chi Minh City, Viet Nam*

*^c^Institute of Biotechnology and Food Technology, Industrial University of Ho Chi Minh City, Ho Chi Minh City, Vietnam*

***Corresponding author:** [dtanhdao@hcmut.edu.vn](mailto:dtanhdao@hcmut.edu.vn) (Dong Thi Anh Dao)

**Abstract**

*Phellinus igniarius*, a medicinal mushroom containing many active ingredients with health benefits, can be applied in functional food. At present, the quantification of the main active ingredients from higher fungi (*Ganoderma*, *Phellinus…)* materials from different growing sources is a mandatory requirement to standardize the input resources of pharmaceutical and food production. Our study’s aims are to perfect the RP HPLC-PDA method for quantitative analysis of Inoscavin A and Meshimakobnol A which are two the main of active ingredients present in *Phellinus* mushroom. In this analytical method, a C18-HPLC column and the mixture of methanol and formic acid solutions (pH=2.2) are used to analyze and elute the active substances with the column activity parameters being the concentration gradient. This perfect method was tested for the system suitability, repeatability, intermediate precision, recovery, and linear curve calibration to validate the method. After validation, the perfected RP HPLC-PDA method was applied to analyze 8 samples of *Phellinus* and 3 samples of *Ganoderma mushroom category*. This method can be the basis for classifying between *Phellinus* and some other medicinal mushrooms.

***Keyword****: Phellinus Spp., Inoscavin A, Meshimakobnol A, RP HPLC-PDA, quantitative analysis.*

**Contents**

[**Experimental for isolating Inoscavin A and Meshimakobnol A purity standard substances** 4](#_Toc142750888)

[**Figure S.1**: Diagram of isolating Inoscavin A and Meshimakobnol A purity standards 5](#_Toc142750889)

[**Identification the structure of Inosavin A and Meshimakobnol A** 6](#_Toc142750890)

[**Figure S.2:** ^1^H NMR spectrum of Inoscavin A 7](#_Toc142750891)

[**Figure S.3:** ^1^C NMR spectrum of Inoscavin A 7](#_Toc142750892)

[**Table S.1.** ^1^H and ^13^C NMR spectral data of Inoscavin A (in Acetone-d6, ^1^H: 500MHz, ^13^C: 125MHz) 8](#_Toc142750893)

[**Figure S.4:** The structure of Inoscavin A 8](#_Toc142750894)

[**Figure S.5:** ^1^H NMR spectrum of Meshimakobnol A 9](#_Toc142750895)

[**Figure S.6:** ^1^C NMR spectrum of Meshimaobnol A 9](#_Toc142750896)

[**Table S.2.** ^1^H and ^13^C NMR spectral data of Meshimakobnol A (in DMSO, ^1^H: 500MHz, ^13^C: 125MHz) 10](#_Toc142750897)

[**Figure S.7:** The structure of Meshimakobnol A 10](#_Toc142750898)

[**Table S.3.** The portrait of *Phellinus spp.* and *Ganoderma spp.* mushoroom 11](#_Toc142750899)

[**REFERENCE** 15](#_Toc142750900)

**Experimental for isolating Inoscavin A and Meshimakobnol A purity standard substances**

The fruiting bodies of *P. igniarius* were collected in Puhuong National Park of Nghean Province, Vietnam in April 2019 and identified by Prof. Dr. Ngo Anh, Department of Biology, Hue University. The purity standard was prepared according to Thanh (2018) [1]. The air-dried and powdered fruit body of *P. igniarius* (11kg) was extracted by 98% EtOH at room temperature for 3 $\times$ 7 days. Then, the extracted was removed solvent by a rotary evaporator (IKA® RV 10, Germany) under temperature of 40^0^C. After removing solvents, the deep brown syrup (700g) was suspended in water and partitioned with ethyl acetate to afford ethyl acetate fractions (56 g).

The ethyl acetate fractions (EAF) were applied to silica gel column chromatography (600g, 160 $\times$ 7 cm) with a chloroform:methanol (C:M) step gradient system (100:0, 40:1: 30:1; 20:1; 10:1: 4:1; 2:1) to obtain 7 minor fractions. This process was supervised by TLC to obtain 5 major fractions. Fraction 3 was eluted into the silica gel column chromatography with the mixture C:M (30:1) to afford Inoscavin A (120mg). The Inoscavin A (120 mg) was purified by HPLC with Luna C_18_ preparative column (methanol:water 20:1; 2.0 mL/min) to obtain inoscavin A (45 mg). Fraction 5 was eluted into the silica gel column chromatography by the mixture C:M (10:1) to get Meshimakobnol A (20 mg). The Meshimakobnol A (20 mg) was purified by HPLC with Luna C_18_ preparative column (methanol:water 4:1 ; 2.0 mL/min) to give Meshimakobnol A (5 mg).

*P.igniarius*

EtOH extract

EtOH, 20L$\times$3

EtOAc fractions

PBE 1

PBE 2

PBE 3

PBE 4

PBE 5

C:M = 30:1

Inoscavin A (120 mg)

Inoscavin A (45 mg)

LC C_18_, M:W = 20:1; 2.0 mL/min

Meshimakobnol A (20 mg)

Meshimakobnol A (5 mg)

LC C_18_, M:W = 4:1; 2.0 mL/min

C:M = 10:1

C:M = 100:0, 40:1: 30:1; 20:1; 10:1: 4:1; 2:1

**Figure S.1**: Diagram of isolating Inoscavin A and Meshimakobnol A purity standards

**Identification the structure of Inosavin A and Meshimakobnol A**

The obtained powders after being eluted into the silica gel column chromatography was identified structure by using Nuclear magnetic resonance spectroscopy (NMR) and compared with the old data reported before in the cited literature. Compound **(1)** has structure similar to Inoscavin A that was described by Kim (1999) [2] and Thanh (2016) [1]. Compound **(2)** has structure similar to Meshimakobnol A that was described by Mo (2004) [3] and Thanh (2018) [1]. The spectroscopic data analysed by NMR was shown below.


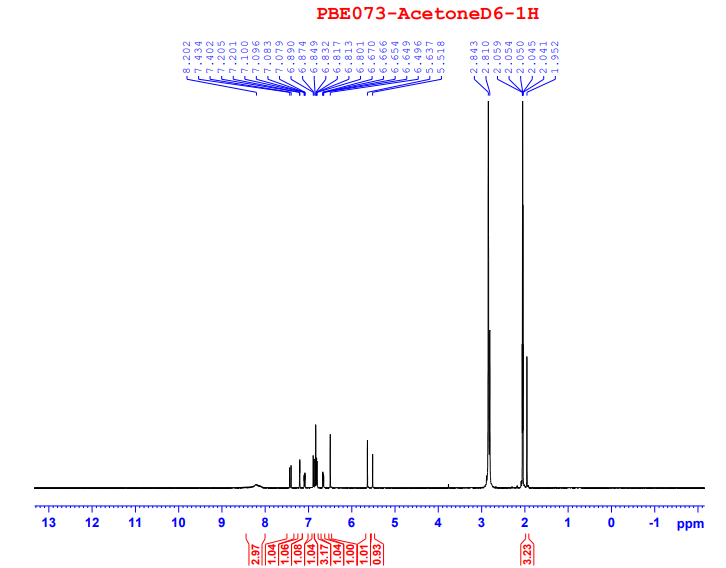


**Figure S.2:** ^1^H NMR spectrum of Inoscavin A


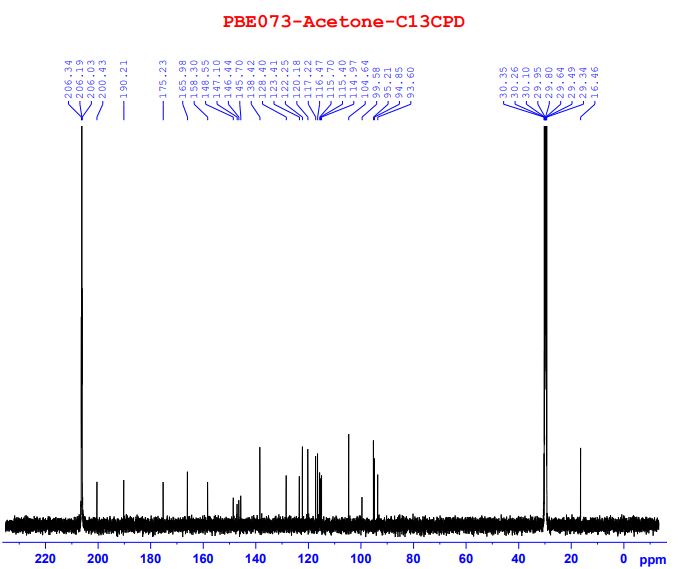


**Figure S.3:** ^1^C NMR spectrum of Inoscavin A

**Table S.1.** ^1^H and ^13^C NMR spectral data of Inoscavin A (in Acetone-d6, ^1^H: 500MHz, ^13^C: 125MHz)

| **Position** | ***δ*^1^H (*J*, Hz)** | ***δ*^1^H**^*^ **(*J*, Hz)** | ***δ*^13^C** | ***δ*^13^C**^*^ | **HMBC - correlations** | **COSY - correlations** |
| --- | --- | --- | --- | --- | --- | --- |
| 1 |  |  | 158.3 | 157.5 |  |  |
| 2 |  |  | 99.6 | 97.9 |  |  |
| 3 |  |  | 175.2 | 174.2 |  |  |
| 4 | 5.64 (s) | 6.71 (s) | 94.9 | 93.5 |  |  |
| 5 |  |  | 166.0 | 164.9 |  |  |
| 6 | 6.82 (d, 15.5) | 6.73 (d, 16.0) | 117.2 | 115.9 | 5, 8 | 6 |
| 7 | 7.42 (d, 16.0) | 7.34 (d, 16.0) | 138.4 | 137.5 | 5, 9, 13 | 7 |
| 8 |  |  | 128.4 | 126.4 |  |  |
| 9 | 7.20 (d, 2.0) | 7.08 (d, 1.5) | 115.0 | 114.6 | 7, 11, 13 |  |
| 10 |  |  | 146.4 | 145.7 |  |  |
| 11 |  |  | 148.6 | 148.2 |  |  |
| 12 | 6.89 (d, 8.3) | 6.79 (d, 8.5) | 116.5 | 115.8 | 8, 10 | 13 |
| 13 | 7.09 (dd, 2, 8.3) | 7.01 (dd, 1.5, 8.0) | 122.3 | 121.2 | 7, 9, 11 | 12 |
| CH_3_ | 1.93 (s) | 1.93 (s) | 16.5 | 16.2 | 1’, 2’ |  |
| 1’ |  |  | 190.2 | 189.8 |  |  |
| 2’ | 5.52 (s) | 5.64 (s) | 104.6 | 103.8 | 1’, 3’, 4’ |  |
| 3’ |  |  | 200.4 | 199.6 |  |  |
| 4’ |  |  | 93.6 | 92.2 |  |  |
| 5’ | 6.50 (s) | 5.66 (s) | 95.2 | 94.4 | 2, 3, 5 |  |
| 6’ |  |  | 123.4 | 121.2 |  |  |
| 7’ | 6.82 (d, 2.3) | 6.67 (d, 2.0) | 115.4 | 114.6 | 4’, 11’ |  |
| 8’ |  |  | 145.7 | 144.9 |  |  |
| 9’ |  |  | 147.7 | 146.4 |  |  |
| 10’ | 6.84 (d, 8.4) | 6.78 (d, 8) | 115.7 | 115.2 | 6’, 8’ | 11’ |
| 11’ | 6.66 (dd, 2.3, 8.4) | 6.54 (dd, 2.0, 8.5) | 120.2 | 118.7 |  | 10’ |

^*^ ^1^H and ^13^C NMR spectral data comparition with Thanh and et al. [4]

**Figure S.4:** The structure of Inoscavin A


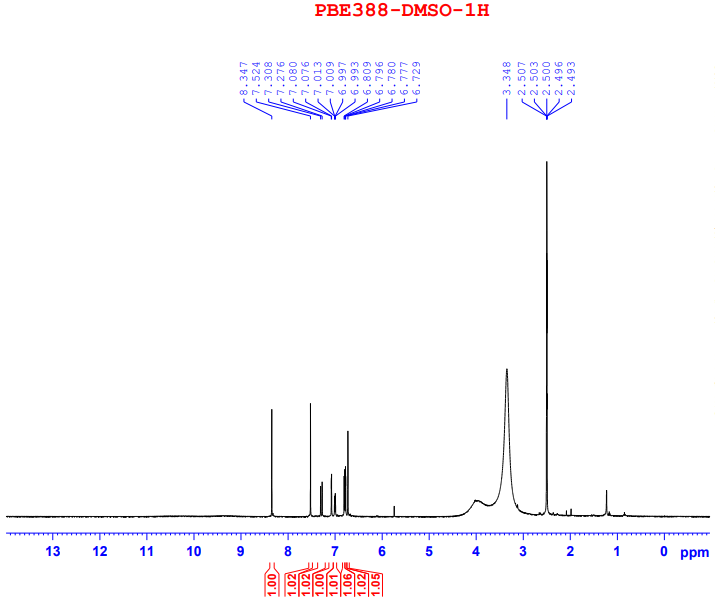


**Figure S.5:** ^1^H NMR spectrum of Meshimakobnol A


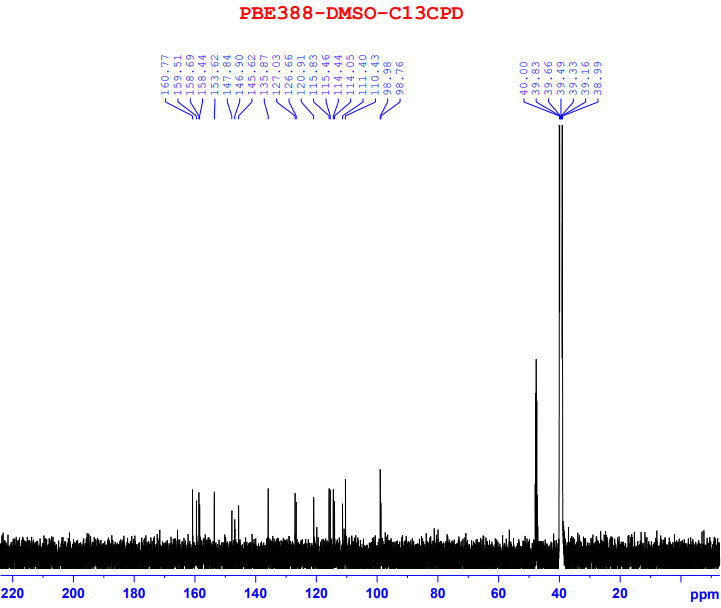


**Figure S.6:** ^1^C NMR spectrum of Meshimaobnol A

**Table S.2.** ^1^H and ^13^C NMR spectral data of Meshimakobnol A (in DMSO, ^1^H: 500MHz, ^13^C: 125MHz)

| **Position** | ***δ*^1^H (*J*, Hz)** | ***δ*^1^H^*^ (*J*, Hz)** | ***δ*^13^C** | ***δ*^13^C^*^** |
| --- | --- | --- | --- | --- |
| 1 |  |  | 159.5 | 159.4 |
| 3 |  |  | 158.4 | 158.4 |
| 4 | 6.73 (s) | 6.66 (s) | 98.9 | 98.9 |
| 4a |  |  | 160.8 | 160.7 |
| 6 |  |  | 158.7 | 158.6 |
| 6a |  |  | 111.4 | 111.4 |
| 7 | 7.52 (s) | 7.51 (s) | 114.4 | 114.4 |
| 8 |  |  | 146.9 | 146.8 |
| 9 |  |  | 153.6 | 153.5 |
| 10 | 8.35 (s) | 8.33 (s) | 110.4 | 110.4 |
| 10a |  |  | 127.0 | 127.0 |
| 10b |  |  | 98.8 | 98.7 |
| 1’ | 6.79 (d, 16) | 6.77 (d, 15.9) | 115.5 | 115.4 |
| 2’ | 7.29 (d, 16) | 7.27 (d, 15.9) | 135.9 | 135.8 |
| 3’ |  |  | 126.7 | 126.7 |
| 4’ | 7.08 (d, 2) | 7.07 (d, 1.5) | 114.1 | 114.1 |
| 5’ |  |  | 145.6 | 145.5 |
| 6’ |  |  | 147.8 | 147.7 |
| 7’ | 6.79 (d, 8) | 6.76 (d, 7.7) | 115.8 | 115.8 |
| 8’ | 7.00 (dd, 2, 8) | 6.99 (dd, 7.7, 1.5) | 120.9 | 120.8 |

^*^ ^1^H and ^13^C NMR spectral data comparition with Mo and et al. [3]


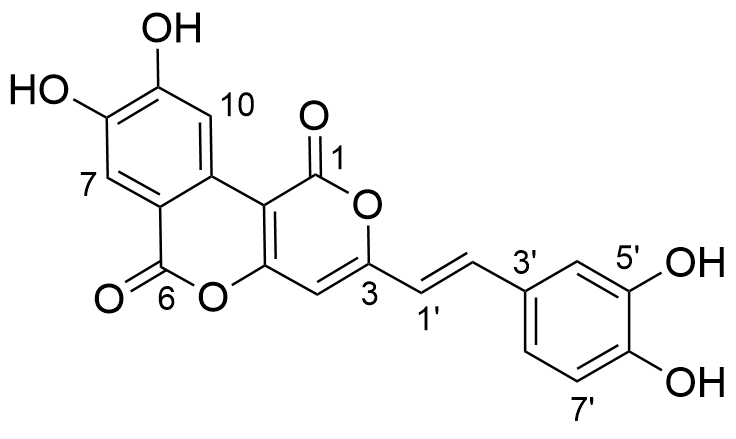


**Figure S.7:** The structure of Meshimakobnol A

**Table S.3.** The portrait of *Phellinus spp.* and *Ganoderma spp.* mushoroom

| **Number** | **Name** | **Image** |
| --- | --- | --- |
| **1** | *Phellinus nilgheriensis* | 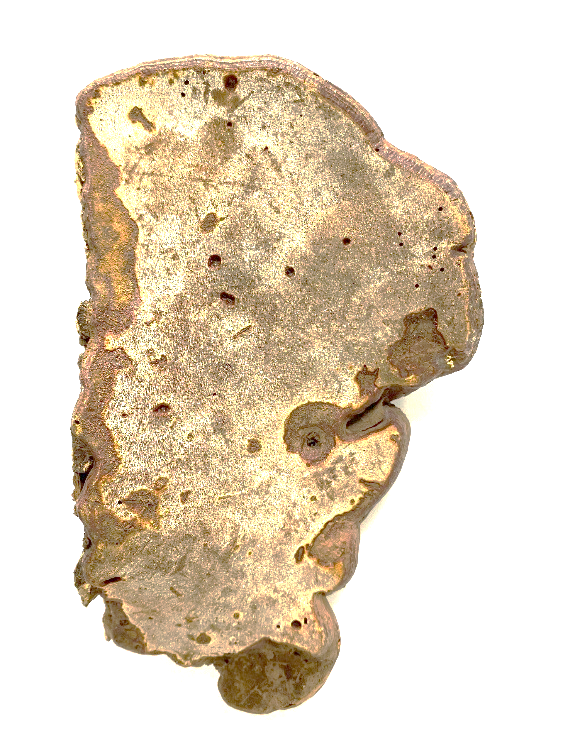 |
| **2** | *Phellinus baumii* | 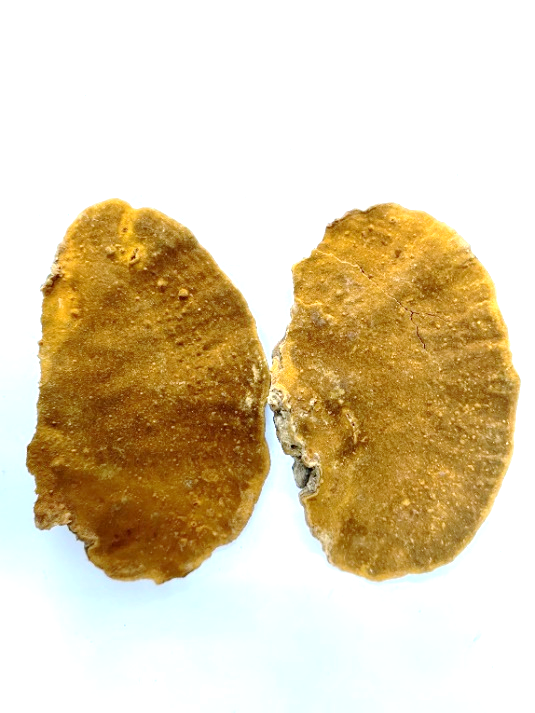 |
| **3** | *Phellinus linteus* | 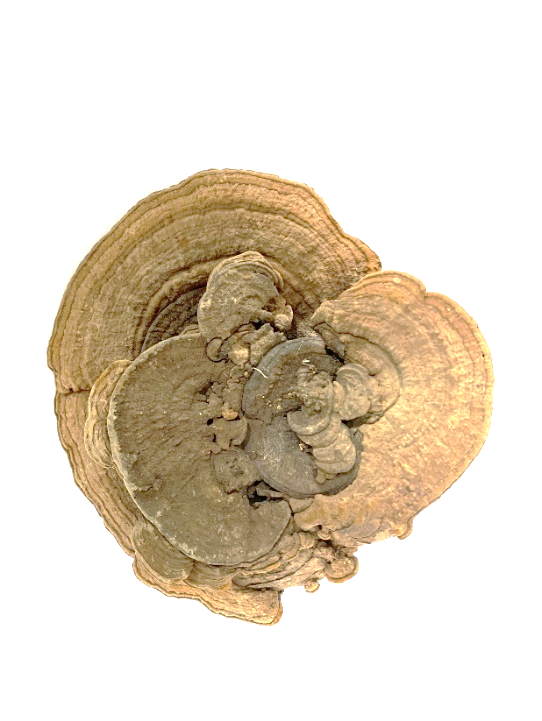 |

| **4** | *Phellinus linteus* | 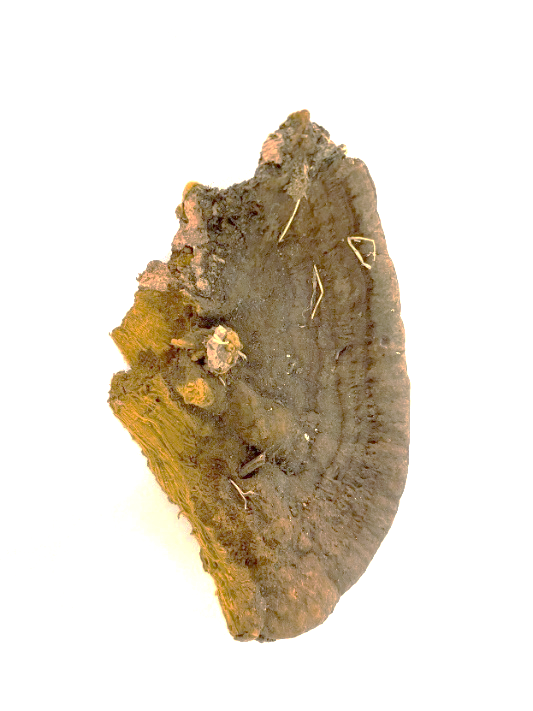 |
| --- | --- | --- |
| **5** | *Phellinus pomaceus* | 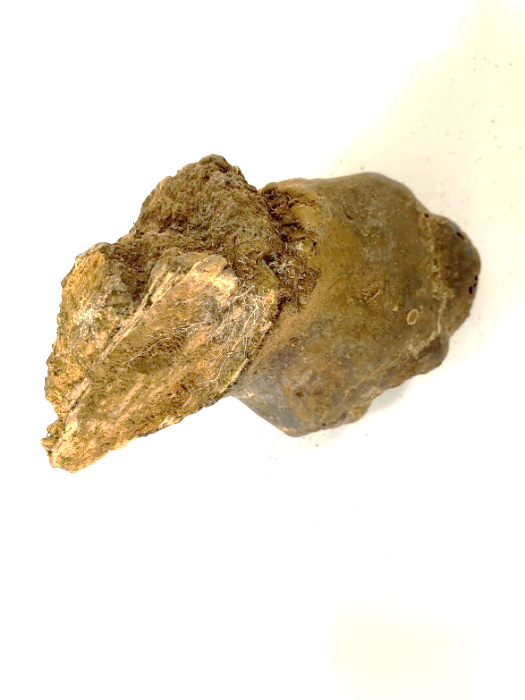 |
| **6** | *Phellinus pini* | 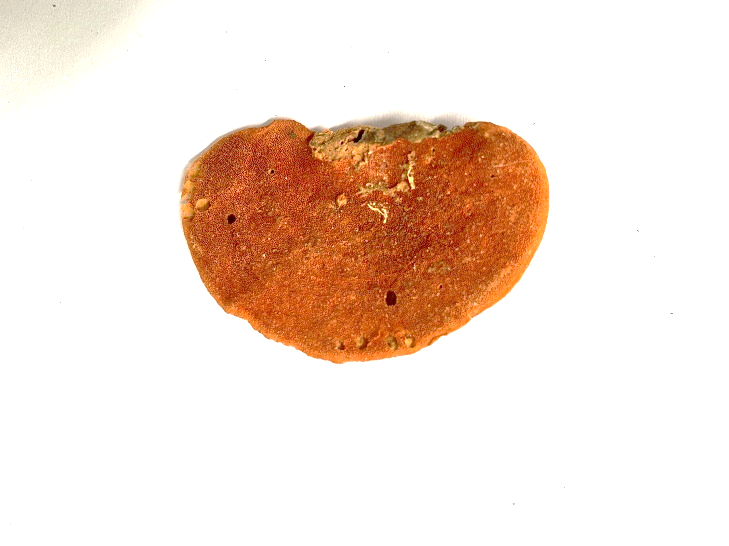 |
| **7** | *Phellinus igniarius* | **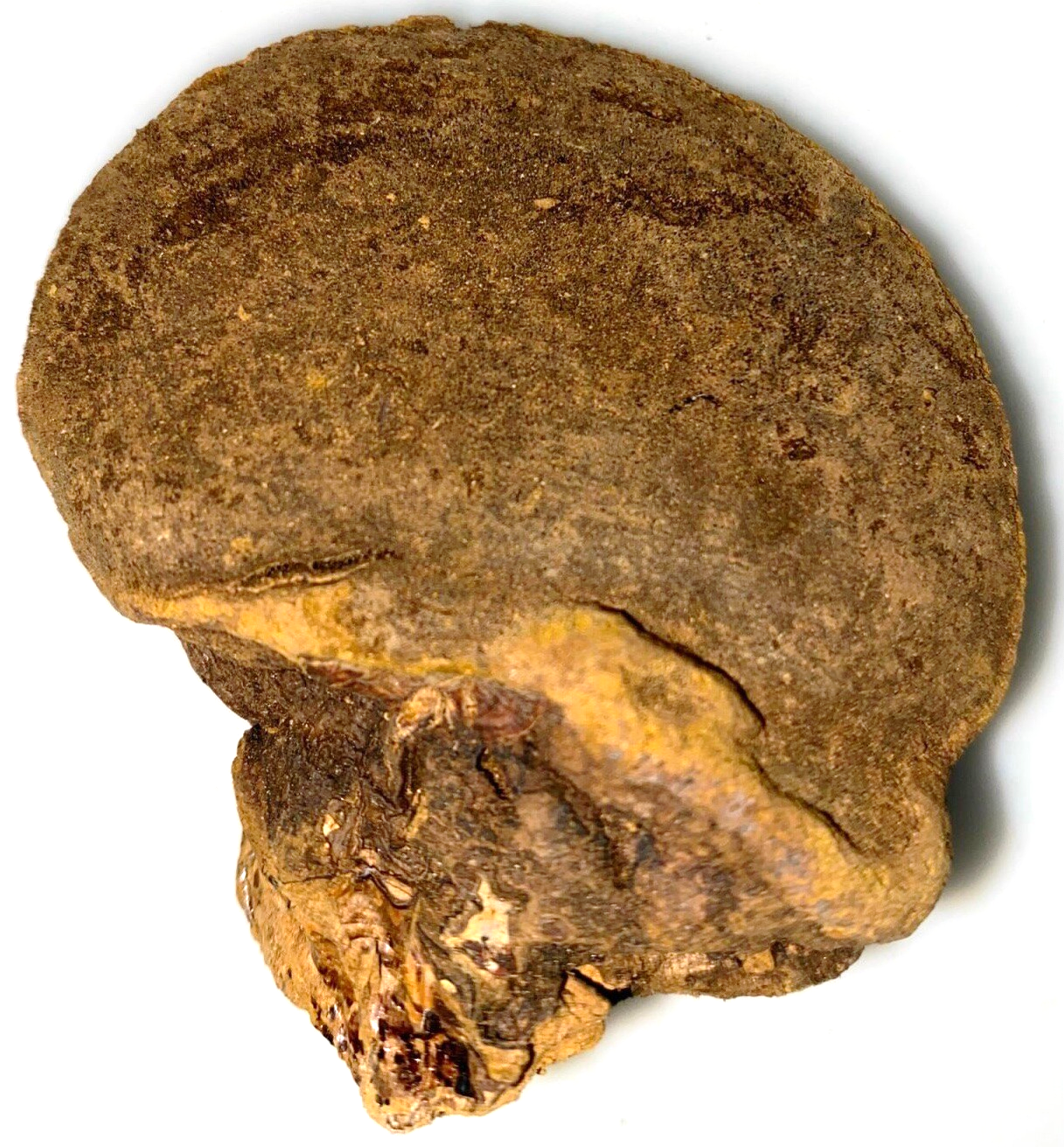** |
| **8** | *Phellinus igniarius* | **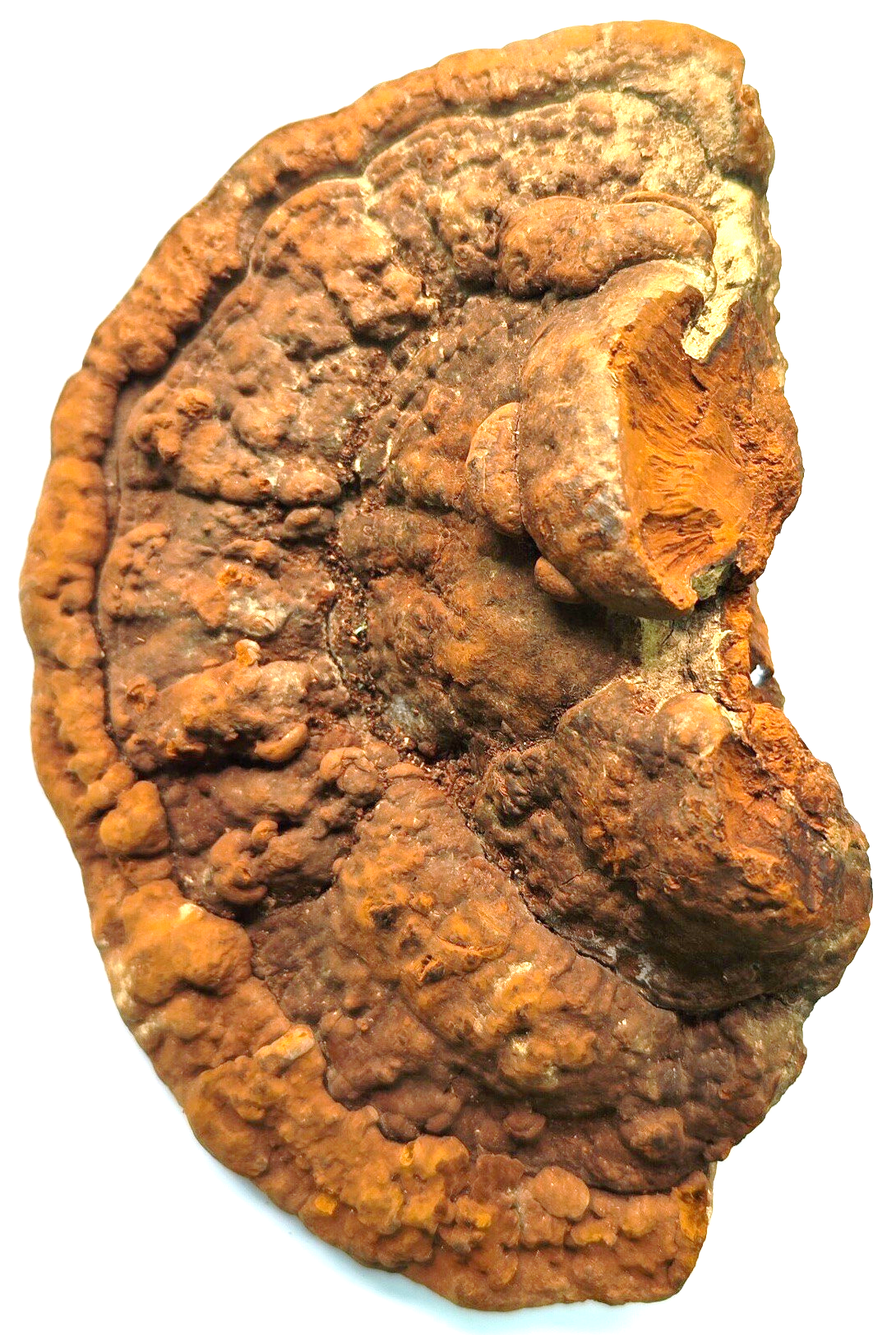** |
| **9** | *Ganoderma applanatum* | 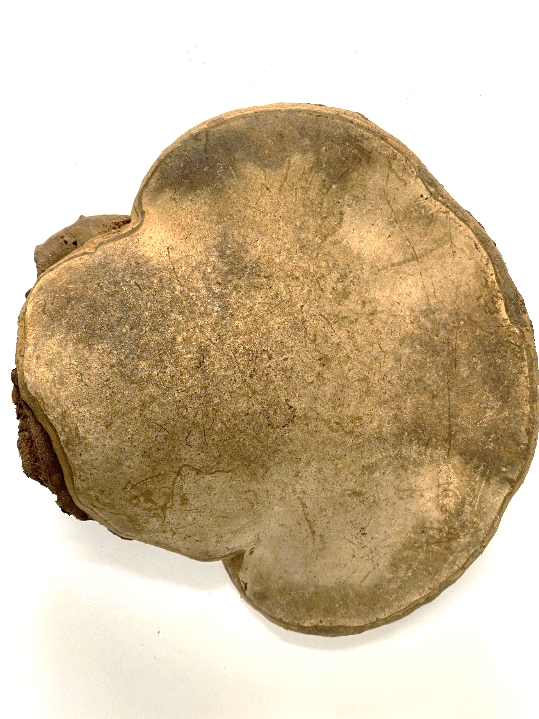 |
| **10** | *Ganoderma australe* | 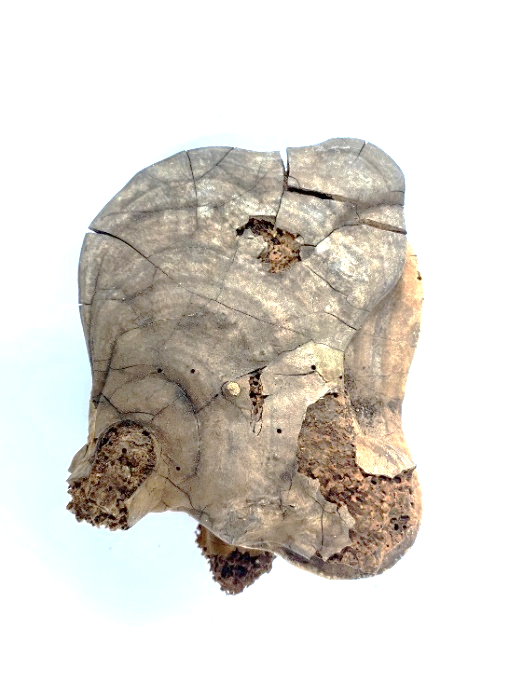 |
| **11** | *Ganoderma brownii* | 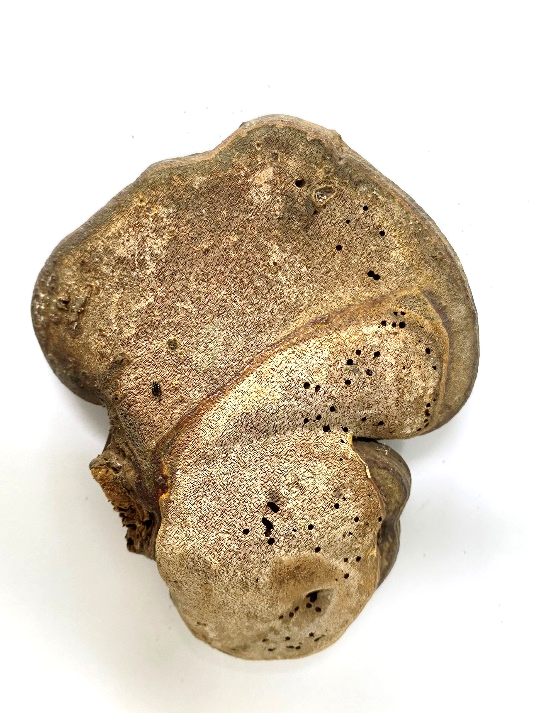 |

**REFERENCE**

1. Thanh, N.T., et al., *Chemical constituents from the fruiting bodies of Phellinus igniarius.* Natural product research, 2018. **32**(20): p. 2392-2397.

2. Kim, J.-P., et al., *Inoscavin A, a new free radical scavenger from the mushroom Inonotus xeranticus.* Tetrahedron Letters, 1999. **40**(36): p. 6643-6644.

3. Mo, S., et al., *Phelligridins C− F: cytotoxic Pyrano [4, 3-c][2] benzopyran-1, 6-dione and Furo [3, 2-c] pyran-4-one derivatives from the fungus Phellinus i gniarius.* Journal of natural products, 2004. **67**(5): p. 823-828.

4. Nguyễn, T.T., et al., *Các hợp chất Phenolic và Steroit từ quả thế nấm thượng hoàng (Phellinus igniarius) ở Việt Nam.* VNU Journal of Science: Natural Sciences and Technology, 2016. **32**(4).
